# A Mobile Health Platform for Heart Failure Self-Management: Feasibility Study on Patient Engagement, Acceptance, and Potential Health Outcomes

**DOI:** 10.2196/89416

**Published:** 2026-07-10

**Authors:** Jane Li, Marlien Varnfield, Andrew A Bayor, Norm Good, Haunnah Rheault, Estelle Beevors, Kathryn Stibijl, Maricel Roxas, Scott McKenzie

**Affiliations:** 1 Australian e-Health Research Centre, CSIRO, Level 7, Surgical Treatment and Rehabilitation Service, 296 Herston Road, Herston, Brisbane, Queensland, 4029, Australia, 61 02 93724163.; 2 Advanced Heart Failure and Transplant Unit, The Prince Charles Hospital, Brisbane, Australia; 3 Cardiology Clinical Research Centre, The Prince Charles Hospital, Brisbane, Australia; 4 PCH Northside Clinical Unit, The University of Queensland, Brisbane, Australia; 5 School of Medicine, The University of Queensland, Brisbane, Australia

**Keywords:** mobile health, heart failure, feasibility study, self-management, patient engagement

## Abstract

**Background:**

Heart failure is a chronic condition that significantly impacts patients’ quality of life and increases health care burden. Effective self-monitoring and lifestyle modification are essential components of heart failure management and can support improved health outcomes. Mobile health technologies, such as smartphone apps, are increasingly used to assist patients with heart failure in self-management. However, evidence regarding patient engagement, user experience, and the effectiveness of these mobile health tools remains limited and continues to evolve.

**Objective:**

This study aimed to explore the feasibility of a mobile health platform, MoTER-HF, which incorporates a smartphone app and a web-based clinical portal to support self-management in patients with heart failure.

**Methods:**

The feasibility study used a single-group pretest-posttest mixed methods design. A total of 23 participants diagnosed with heart failure were recruited to use the app and 2 Bluetooth-enabled measurement devices (a blood pressure monitor and a digital weight scale) over a 12-week period. Participants’ engagement and acceptance were assessed using a satisfaction questionnaire, semistructured interviews, and platform usage logs. Potential health and behavioral outcomes were explored using validated instruments administered at baseline and week 12.

**Results:**

Most participants found the MoTER-HF app easy to use and aligned with their routine self-monitoring practices. Daily monitoring features such as blood pressure and weight tracking were used frequently. However, features such as symptom tracking and exercise logging were used less often, reflecting individual preferences and perceived relevance. Participants reported improved self-monitoring practices and valued the ability to visualize and track their data, and the reassurance provided through nurses’ oversight in the satisfaction questionnaire and interviews. Changes in health and behavioral outcome measures were not statistically significant, although exploratory changes were observed in the scores of self-care, quality of life, and psychological well-being.

**Conclusions:**

The MoTER-HF platform has demonstrated potential in supporting self-management among individuals with heart failure, particularly when it incorporates features that participants find engaging. Further research is needed to better understand the platform’s impact on health outcomes and the implementation challenges, and to involve clinicians in developing a scalable digital model of care.

## Introduction

### Background

Heart failure is a complex health problem characterized by debilitating symptoms, which adversely affect morbidity [1,2]. Despite advances in medical, pharmacological, and surgical treatments, the global prevalence of the condition continues to rise, and hospital admissions remain high [3,4]. Heart failure significantly impacts patients’ quality of life (QoL) and contributes to increasing health care expenditure [5,6]. Evidence suggests that individual behavior change and community support can help reduce hospitalizations related to heart failure [7-9]. Self-management support has become a critical strategy to empower patients with heart failure to actively engage in and take responsibility for their health and care [2,8,10,11]. Patients are encouraged to adopt healthier lifestyles and strengthen self-management behaviors, including symptom monitoring, physical activities, fluid management, and treatment adherence [8,12].

Mobile health (mHealth) technologies, such as smartphone apps, internet-based tools, wearable devices, and other patient monitoring systems, are increasingly being used to support the self-management of patients with chronic cardiac diseases [13,14]. Early evidence from experimental studies and randomized controlled trials (RCTs) involving patients with heart failure suggests that mHealth interventions can improve disease knowledge, self-care, and QoL [15-19]. However, most of these RCTs have been primarily feasibility studies or small-scale trials, and the clinical, health, and behavioral outcomes reported have been inconsistent, highlighting the need for further investigation [18,19].

Research on the design of mHealth technologies for heart failure self-management is also evolving [16,18,20]. Existing mHealth apps vary widely in the features they offer, with some lacking key components recommended by clinical guidelines and patient empowerment strategies, such as symptom monitoring, physical activity support, and patient education [18,19]. Digital interventions based on mHealth technology need to be adapted to the local context and involve both patients and clinicians in the design process [21]. To enhance effectiveness and promote patient empowerment, these interventions need to be more tailored and to incorporate features associated with improved clinical and behavioral outcomes [18,22-24].

Patient engagement with mHealth interventions remains a challenge [23]. Understanding patients’ usage behavior, motivation, and preferences in response to the interventions is crucial for comprehending how patients with heart failure interact with the interventions and for assessing their level of acceptance [22,24,25]. Measures of engagement vary across studies of mHealth [20]. Combining system usage data with qualitative insights on engagement patterns and quantitative outcome assessments may provide a more comprehensive understanding of acceptability and impact [18,20]. Given the importance of patient preferences and sustained engagement, evaluation studies need to examine not only health outcomes but also influencing factors for engagement [22].

### The MoTER-HF Platform

**Figure 1.**
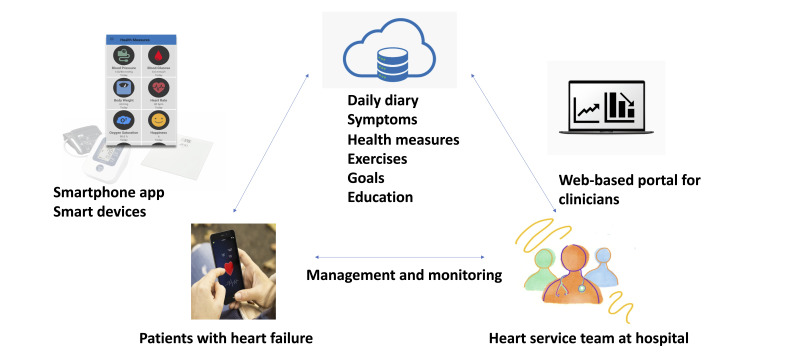
Structure and components of the MoTER-HF platform.

An mHealth platform was developed through a collaboration between the Commonwealth Scientific and Industrial Research Organization (CSIRO) and The Prince Charles Hospital (TPCH) in Australia to support patients with heart failure. The mHealth platform, MoTER-HF, was developed from a research prototype called MoTER (Mobile Technology–Enabled Rehabilitation), which was originally designed for home-based monitoring in cardiac rehabilitation [14]. The platform incorporated a smartphone app and a web-based clinical portal (Figure 1). The clinical portal enabled clinicians to create patient accounts and configure personalized health measurements and goals. Patient-reported health measurements, symptoms, and exercise data collected through the app were automatically uploaded to the portal, allowing clinicians to monitor the data. The app stored data locally and synchronized it with the portal whenever a data connection was available.

The initial MoTER-HF prototype was refined after being tested by patients and clinicians from TPCH to tailor the features to the needs of patients living with heart failure. During the user testing, 9 patients with a clinical diagnosis of heart failure installed and tested the app for 2 weeks, while 8 clinicians (2 cardiologists, 1 nurse practitioner, 1 clinical nurse consultant, 2 cardiac nurses, 1 physiotherapist, and 1 pharmacist) reviewed the app and tested the clinical portal in design-oriented workshop sessions. On the basis of their feedback regarding functionality and clinical relevance, the study team identified areas for improvement, addressed technical issues, and refined the app features. Key app refinements resulting from user testing included the development of the daily self-monitoring questions by clinicians in accordance with clinical guidelines [2], the addition of a text message on the Symptoms page prompting patients to seek medical care if symptoms deteriorated, revisions to the symptom list and exercise list for patients to check, and an updated set of web-based educational resources.

The final version of the MoTER-HF app (available on iOS and Android) included the following features (Textbox 1; see also Multimedia Appendix 1).

Textbox 1. **Key features of the MoTER-HF app.**

**Health Measures**

 Patients could record and check daily health measure data, such as blood pressure (BP), weight, blood glucose, and other health measures customized by clinicians. Two Bluetooth-enabled monitoring devices (a BP monitor and a digital weight scale) were provided by the study team. These devices could be paired with the app to enable automatic data capture of BP, heart rate, and weight, with data uploaded directly to the clinical portal.

**Daily Diary**

 Patients answered 4 questions related to the heart failure signs and symptoms, such as weight gain; the need for extra pillows during sleep; shortness of breath during physical activity or at night; and swelling in feet, ankles, legs, or abdomen. A daily app notification was sent to patients as a reminder for diary completion.

**Symptoms**

 Common symptoms (eg, cough, rapid heartbeat, chest discomfort, heaviness, and loss of appetite) were listed for patients to check and record details if experienced.

**Exercises**

 Ten common exercises (eg, walking and gym) were listed for patients to log and rate exertion using the Borg scale. 

**Education**

 Resources on heart failure management, healthy eating, and holistic well-being were provided, with a link to the National Heart Foundation of Australia website. 

**Goals**

 Lifestyle modification and health monitoring goals could be set with guidance from clinicians.   

The platform was evaluated in a feasibility study involving a cohort of participants diagnosed with heart failure. The study aimed to assess the feasibility of the app among these patients and explore potential health outcomes associated with the app use.

## Methods

### Study Design

The feasibility study used a single-group pretest-posttest mixed methods design. The study examined the feasibility of the app, including patients’ adherence, acceptability, and perceived usability, identified factors influencing patient engagement and acceptability, and explored potential health outcomes. Feasibility outcomes were assessed descriptively without predefined benchmarks or progression criteria and were treated as the primary outcomes of the study. The investigation of health and behavioral outcomes was exploratory and not an effectiveness evaluation of the intervention. The study followed the International Council for Harmonization’s Good Clinical Practice guidelines to ensure the protection of participants’ rights and provide assurance for data integrity.

### Feasibility Study Participants

Feasibility study participants were recruited from the TPCH Heart Failure Service between April 2024 and November 2024. Eligibility criteria included (1) adults (aged >18 y) with a primary or secondary diagnosis of heart failure or outpatients who had been hospitalized for heart failure in the last 12 months, (2) proficient in English, (3) had access to a smartphone compatible with the MoTER-HF app, (4) the ability to operate a smartphone for the purposes of the study (eg, no vision, hearing, and cognitive or dexterity impairments), and (5) access to mobile internet and/or Wi-Fi at home. Patients who were unable to attend TPCH Heart Failure Service or were involved in another intervention study were excluded.

### The Intervention Procedures

A clinical nurse coordinator assisted participants with installing the MoTER-HF app, configuring the health measures to be tracked, and discussing suitable goals following consultation with the treating clinician during the onboarding visit. Participants were provided with 2 Bluetooth-enabled measurement devices (a blood pressure [BP] monitor and a digital weight scale) and used the app and devices over a 12-week study period. During this time, they were required to log Health Measures and Daily Diary responses via the app on a daily basis, log Symptoms and Exercises when applicable, and use the Education and Goals when needed. Training on using the app was provided by the nurse coordinator during participants’ onboarding visits. Participants also received a printed user manual outlining the app’s function, step-by-step instructions, and contact details (phone and email) of the hospital’s cardiac clinical research center for technical support or study-related questions.

A nurse practitioner, who was also an investigator of the study, discussed the app data with participants during in-person clinic consultations as part of routine care. Although clinicians had access to participant data at any time, continuous monitoring was not performed in the feasibility study due to workload constraints. Instead, the clinical nurse coordinator reviewed the data twice weekly. Thresholds for each measure were set up by the nurse coordinator in the clinical portal. Alerts for out-of-range values were flagged in the portal, and email notifications were automatically sent to the TPCH Heart Failure Service for the nurse coordinator to follow up by phone. The nurse coordinator also monitored participants’ adherence to app use and provided technical support to participants who experienced difficulties using the app.

### Outcome Measures and Data Collection

The primary outcomes of this study were feasibility measures, which were assessed as adherence, usability, and acceptability (Table 1).

**Table 1.** Feasibility domains, measures, data, and analysis^a^.

Feasibility domain	Description of measures	Data and analysis
Adherence	Adherence was assessed by participants’ *engagement* with the MoTER-HF app over their 12-week study period, characterized by frequency of use, consistency of use, and breadth of use.	Quantitative analysis of app logs exported from the platform server (eg, number of data entries for each app feature) Quantitative analysis of satisfaction questionnaire responses related to frequency of use, engagement, and perceptions of app features Qualitative analysis of interview data examining participants’ patterns of use across app features, interaction experience, and factors influencing engagement
Usability	Usability was assessed by participants’ perceptions of the usability, user interface, and functionality of the app features.	Quantitative analysis of System Usability Scale and satisfaction questionnaire responses related to user interface design Qualitative analysis of interview data related to usability and interaction experience with the app features
Acceptability	Acceptability was assessed by participants’ perceptions of the app’s compatibility with their needs and daily routines, perceived empowerment experience, their observability, and overall satisfaction.	Quantitative analysis of the satisfaction questionnaire items addressing empowerment, observability, and overall satisfaction Qualitative analysis of interview data explored perceived benefits and factors influencing acceptability

a Engagement (operationalized as adherence in this study) was defined as participants’ interaction with the intervention over time, characterized across the dimensions of frequency of use (eg, number of feature interactions), consistency of use (eg, regularity of engagement across the study period), and breadth of use (eg, range of features used).

The feasibility measures were investigated through a satisfaction questionnaire, semistructured interviews, and MoTER-HF app usage logs. The satisfaction questionnaire (Multimedia Appendix 2) was developed by the research team based on the Diffusion of Innovation Theory [26] and our previous studies [27,28]. It includes engagement questions related to frequency of use, perceptions of each app function, and compatibility with participants’ daily routines. It also included questions to understand their empowerment experience, its observability, and their overall acceptance of the app. The questionnaire also included the System Usability Scale [29] to assess the subjective usability of the app and questions related to the frequency of use. It was administered after participants completed the 12-week study, within a 2-week poststudy window. App usage logs were exported from the platform server after study completion to assess adherence.

A purposively selected sample of participants, based on their demographic characteristics (eg, sex and age) and level of engagement with the app (eg, frequent or infrequent use according to app usage log), was invited to participate in the qualitative arm for an interview conducted upon their study completion and scheduled to coincide with a clinic visit. The interviews aimed to explore participants’ experiences and perceptions of using the MoTER-HF app. The interviews were conducted by 2 researchers (JL and AAB) via Microsoft Teams video conferencing. Participants attended the interviews from a private office during their end-of-study visits to the hospital, with video conferencing facilities arranged by the nurse coordinator. The 2 researchers conducted debrief discussions after each interview and collectively determined the data saturation. Each interview lasted approximately 30 minutes and was recorded using Microsoft Teams.

Health and behavioral outcomes were measured at baseline and at the 12-week time point, as an exploratory investigation. The following questionnaires were included:

- Self-reported self-care, assessed using the Self-Care of Heart Failure Index (version 7.2) [30]
- QoL, assessed using the Kansas City Cardiomyopathy Questionnaire [31]
- Heart failure knowledge, assessed using the Dutch Heart Failure Knowledge Scale [32]
- Self-efficacy, assessed using the Self-efficacy for Managing Chronic Disease Scale [33]
- Depression, anxiety, and stress, assessed using the 21-item Depression, Anxiety, and Stress Scale [34]

Individual participants were clinically assessed by study clinicians at baseline and at the 12-week time point. Assessments included clinical examinations of height, weight, lying and standing BP, heart rate (HR), respiratory rate, and oxygen saturation, and related physical examinations. A demographic survey was completed by participants at baseline. Major Adverse Cardiovascular Event data were collected from hospital information systems by the clinical nurse coordinator at the 12-week time point.  

All questionnaires were paper-based and collected by the clinical nurse coordinator, who also entered the responses into the secure web-based REDCap platform hosted by the CSIRO.

### Data Analysis

Descriptive statistical analysis was conducted for the satisfaction questionnaire, as well as for app log data, using Microsoft Excel. Changes in health and behavioral outcomes from baseline to 12 weeks were assessed using a 2-tailed paired-samples *t* test in the R environment.

Interview audio recordings were transcribed by a professional transcript service. A thematic analysis was conducted by 2 researchers (JL and AAB) using NVivo software (version 14; Lumivero). Initial codes were developed based on the categories of engagement experience and perceived impact. Coding was cross-checked and discussed between the 2 researchers prior to the second round of coding and results synthesis. Member checking was not performed due to logistical difficulties in reaching participants after the study. Triangulation was used to integrate quantitative usage data (to quantify behavioral interaction patterns), questionnaire responses (to capture perceived engagement and satisfaction), and qualitative interview data (to contextualize behaviors and identify underlying mechanisms).

### Ethical Considerations

Ethical approval was granted by the Metro North Hospital and Health Service Human Research Ethics Committee (project ID: HREC/15/QPCH/256), and reciprocal approval was obtained from the CSIRO Health and Medical Human Research Ethics Committee (project ID: 2021_059_RR). Written informed consents were obtained from all participants. All questionnaire responses and platform log data were stored on secure servers of CSIRO and were identified only through study IDs. Interview transcripts were deidentified before analysis, and the results were reported in aggregate. Participants did not receive compensation for participating in the study.

## Results

### Participant Characteristics

**Figure 2.**
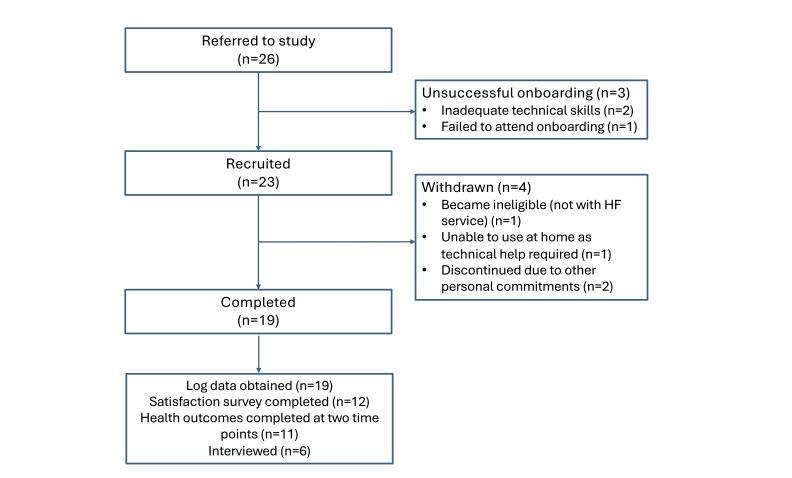
Flow diagram of study participants. HF: heart failure.

A total of 26 eligible candidates were referred to the study team, of whom 23 (88.5%) completed the baseline assessment and were onboarded (Figure 2). The remaining 3 (11.5%) did not complete onboarding due to inadequate technical skills or failure to attend the onboarding visit. Among the 23 participants who installed the MoTER-HF app, 1 (4.3%) withdrew after 3 weeks due to being ineligible (no longer receiving care from TPCH Heart Failure Service), 1 (4.3%) required technical assistance and was unable to use the app at home, and 2 (8.7%) discontinued due to competing personal commitments. Participants’ baseline and end-of-study visits were aligned with their hospital clinical appointments for their convenience. However, difficulties in reaching some patients and coordinating with their clinic schedules at times affected the timeliness of follow-ups and questionnaire data collection. In total, 19 participants completed the study and used the app, and their available data were included in the data analyses. A total of 6 (31.6%) participants took part in the qualitative arm and were interviewed.

The participant cohort (N=19) was primarily men (n=14, 73.7%), with most participants aged 50 to 60 years (n=9, 47.4%) or 40 to 50 years (n=4, 21.1%). The majority (n=15, 78.9%) lived with a partner or children. Most participants had been diagnosed with heart failure within the past year (11/17, 64.7%) and were enrolled in a hospital-based heart failure program (12/19, 63.2%). Table 2 summarizes the key characteristics of the participant cohort. Among the 6 interviewed participants, 4 were men, and 2 were women, with 3 aged 50 to 60 years, 2 aged 60 to 70 years, and 1 aged <50 years.

**Table 2.** Characteristics of feasibility study participants.

Characteristics	Values (N=19), n (%)
Sex	
Male	14 (73.7)
Female	5 (26.3)
Age group (y)	
<50	4 (21.1)
50‐60	9 (47.4)
60‐70	4 (21.1)
70‐80	2 (10.5)
Lives	
Alone	4 (21.1)
With partner and/or children	15 (78.9)
Participating in a heart failure program	
Yes	12 (63.2)
Years since heart failure diagnosed (n=17)	
<1	11 (64.7)
1‐2	5 (29.4)
5	1 (5.9)

### Usability

The usability perception measured by the System Usability Scale was positive (n=12), with an average score of 69.38 (SD 22.39), corresponding to a grade of “good.” Seven (58.33%) participants scored usability above 68, giving the app a Good or Excellent usability rating. 

Interview participants also reported positive experiences with the app’s usability and user interface. They found the app easy to use, especially its simple design and straightforward functionality. Most participants described the app as user-friendly, requiring minimal instructions, and easy to navigate without external assistance:

*It wouldn't turn you off using it because it was so simple.*

[Participant ID5]

To further explore which app features participants valued most, an open-ended question was included: “What did you like best about the app?” A total of 8 (66.7%) participants provided responses, with comments highlighting the aspects of the graphical displays for reviewing BP and weight data (n=2), ease of use (n=2), and Bluetooth integration (n=2). Other mentioned aspects included having all data consolidated in one app (n=1), the ability to share progress (n=1), the simplicity of daily recording (n=1), and reminders (n=1). The majority of the interview participants indicated that the daily input and graphic display of BP and weight data were particularly useful, as they allowed participants to visually track health measurements over time:

*I seem to have lower blood pressure like in the 90s. I could also look at it and think oh it’s not the first time this week I’ve been at 92... It would just give you more confidence that... you were at 120 and now you’re all of a sudden 90. You would look across your percentages and think...it was easier to work out.*

[Participant ID5]

### Adherence

#### Usage Log

BP, HR, and weight were included in each patient’s health measure list configured by the clinical nurse coordinator for daily check-in. Usage logs from the 19 participants showed that all entered their BP and HR data, with a total of 909 entries (mean 47.8, SD 44.4, range 2‐190 over 12 wk). Weight data were entered 757 times by 19 participants (mean 39.8, SD 32.2, range 2‐90 over 12 wk). Notes from the study team’s communication with patients indicated that among the 6 participants with fewer than 20 BP or HR entries, 2 experienced login technical issues, 2 entered their readings manually into the app, but the data failed to synchronize to the online web-based portal due to home Wi-Fi issues, and 2 were not interested in daily recording.

On the basis of participants’ medical conditions, a few had additional health measures included in the app. Blood glucose monitoring was added for 1 participant, resulting in 23 recorded entries, while smoking status was tracked for another participant, with 4 entries recorded.

A total of 16 (84.2%) participants used the Daily Diary feature and answered the 4 questions each day, completing it a total of 236 times (mean 14.8, SD 22.3, range 1-73 over 12 wk). Symptoms were reported by 4 (21.1%) participants (one each), including chest discomfort (n=4), heaviness (n=2), rapid heartbeat (n=1), and cough (n=2). Exercises were logged by 4 (21.1%) participants (2‐28 entries each), mainly walking, home exercise, and gym activities. Table 3 summarizes the count of entries recorded by participants.

**Table 3.** Number of entries recorded by participants from the usage log.

Event name	Participants reported, n (%)	Total count, N	Count per participant, mean
Health Measures—BP^a^ and HR^b^	19 (100)	909	47.8
Health Measures—weight	19 (100)	757	39.8
Daily Diary	16 (84.2)	236	14.8
Symptoms	4 (21.1)	4	—^c^
Exercises	4 (21.1)	64	—

a BP: blood pressure

b HR: heart rate.

c Not applicable.

#### Feature Usage and Engagement

To understand how participants engaged with the app, we examined their self-reported frequency of use in conjunction with their overall perceptions of the app functions, as recorded in the satisfaction questionnaire (n=12). These findings, together with interview results reported in the next 2 subsections (Engagement and User Experience Facilitators and Engagement and User Experience Barriers), provide insights into which features were integrated into their daily routines and which were less frequently adopted.

**Figure 3.**
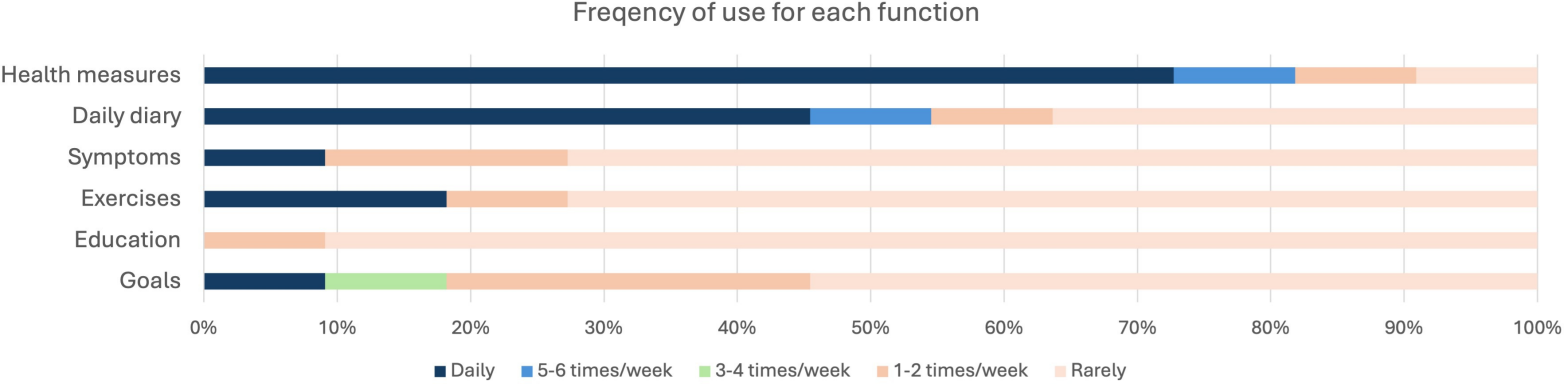
Self-reported frequency of use in the satisfaction questionnaire.

The Health Measures feature was the most consistently used, with the majority of participants (9/11, 81.8%) reporting use either daily or more than 5 to 6 times per week. More than half (6/11, 54.5%) also engaged with the Daily Diary at a similar frequency. A small proportion reported regular use of the Symptoms (1/11, 9.1%) and Exercise (2/11, 18.2%) functions, while the Education and Goals features were rarely accessed (Figure 3).

**Figure 4.**
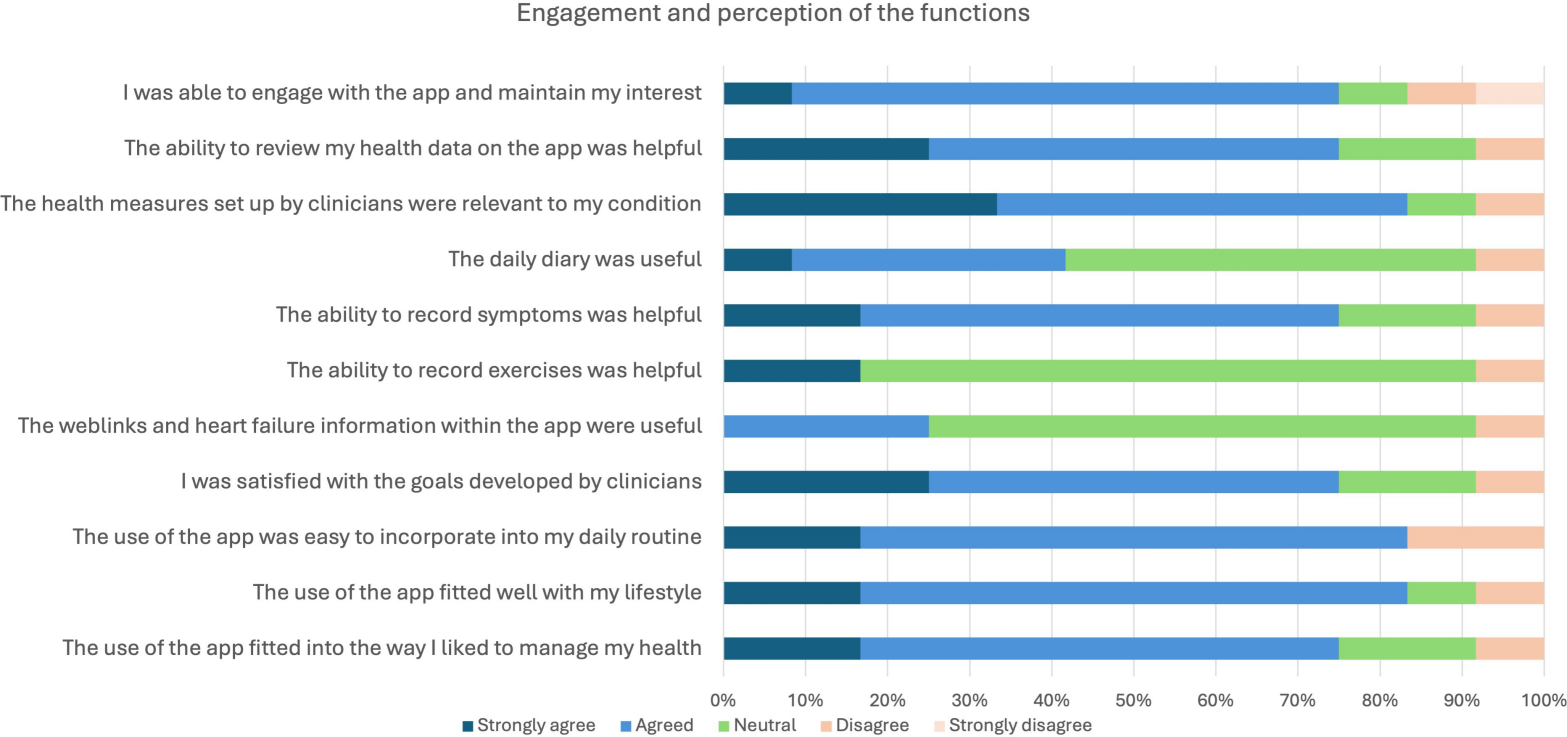
Self-reported engagement and perceptions.

The majority of the participants reported that using the app was easy to incorporate into their daily routines (10/12, 83.3%), fitted well with their lifestyle (10/12, 83.3%), and aligned with the way they liked to manage their health (9/12, 75%; Figure 4). Results also showed that most participants (9/12, 75%) were able to maintain interest in entering and reviewing health data. However, features such as the Exercise tracking and Education information were perceived as less useful. Despite checking infrequently, most participants (9/12, 75%) were satisfied with the clinician-set goals.

#### Engagement and User Experience Facilitators

Interview participants identified several factors that supported their engagement with the app, including alignment with daily routines, reduced burden of use, and enhanced reflection on health progress.

##### Integration With Existing Daily Routines

Engagement was reinforced by preexisting health monitoring habits. For some participants, the transition from paper-based to digital recording was seamless:

*I had to record my weight and symptoms every day in a booklet...so it just went from manual to digital. I was already doing it, so it wasn’t any hardship.*

[Participant ID2]

##### Visual Design and Interactive Appeal

Interview findings echoed responses from the satisfaction questionnaire, with participants highlighting the app’s visual presentation of data, particularly the health measure data. Graphs and color-coded icons (eg, red for out-of-range values) were described as intuitive and engaging:

*It wasn’t just a list of numbers...you could slide backwards and forwards to see trends.*

[Participant ID18]

##### Reflection and Progress Tracking

For most participants, the availability of longitudinal data supported routine tracking, provided reassurance and motivation, and enabled reflection on health progress and pattern recognition:

*I like to be able to see the graph to give me a goal and where it’s heading...you go oh this happens every day or once a week. It gives me more peace of mind that I’m heading in the right direction.*

[Participant ID5]

##### Support for Automatic Data Capture

Participants highlighted the value of Bluetooth syncing for automatic upload of BP, HR, and weight data from the 2 measurement devices. This feature reduced the burden of manual entry, minimized entry errors, and encouraged sustained engagement:

*“It wouldn’t turn you off using it because it was so simple...with the Bluetooth it does it for you. People would stop doing it if it wasn’t this easy.”*

[Participant ID5]

### Engagement and User Experience Barriers

While results of usage log satisfaction questionnaire showed less frequent use for some functions (eg, Symptoms, Exercises, and Education; Table 3 and Figure 3), factors that limited their engagement were discussed in the interviews. These influencing factors included perceptions of limited need and relevance, stable health condition, personal preferences for recording information and learning, and low digital literacy.

#### Perceived Needs and Relevance

Some participants felt that features such as the Symptoms and Daily Diary were useful but not necessarily relevant to their personal needs, as they were in relatively stable condition or already in regular contact with clinicians:

*Just me personally...I was coming to the hospital a lot...I just don’t think that was necessary for me...maybe for other people that get different symptoms and that they might have more questions. But being a diabetic all my life and talking to doctors...I know what I’m doing.*

[Participant ID5]

Similarly, exercise tracking was underused, with participants citing their low activity levels, lack of relevance to their treatment, or redundancy with their existing rehabilitation programs:

*I go to the cardiac gym but I wasn’t using it [the app] to monitor...maybe down the track eventually it could be linked in with it or whatever but at the moment it’s all paper based.*

[Participant ID1]

#### Health Condition and Motivation

Some participants did not use the Symptoms feature or were less motivated to use the Goals feature simply because they experienced no noticeable changes in their conditions during the study:

*I recorded probably half a dozen honestly probably 6 all up over the period because I didn’t really have anything drastically change over the time.*

[Participant ID24]

#### Event Based Rather Than Routine Record

While opening Daily Diary regularly, some participants indicated that they only recorded their answers to the questions when changes in their health occurred for notetaking:

*When something came up it was a good way to quickly make a note...when weighing myself or doing blood pressure. I didn’t record everything each day, but it was a good way to track events.*

[Participant ID24]

#### Reporting and Learning Preferences

Direct interaction with clinicians was preferred over app-based recording or education materials:

*At the time I was talking to heart surgeons...you can’t get much better advice from an app than a heart surgeon.*

[Participant ID5]

Low use of the Education feature was also attributed to limited interest in reading, difficulty engaging with text-based content, a preference for audio-based formats, or concerns about information overload:

*...the fastest way to put me to sleep is give me a text. I’m more auditory, I prefer to listen to what people recommend.*

[Participant ID2]

#### Digital Literacy and Technical Concerns

Several participants with lower digital literacy required technical assistance from family members or the nurse coordinator, especially during the initial phase of the study. One interview participant suggested incorporating a troubleshooting or self-help feature within the app to support users encountering technical difficulties.

While Bluetooth data capture was intended to enable automatic data capture, its use was affected by the reliability of Bluetooth device connections and pairing, as reported by some participants in the satisfaction questionnaire and interviews. These issues led to participant frustrations and prompted a switch to manual data entry, which was a smoother option. Some interview participants also described the feature of daily reminders for data entry unnecessary and “frustrating”

*Because I’m a diabetic I get up every morning. I have my insulin. I weigh myself—you don’t have to remind me... So yes, for some people the reminder is good, but for me it was unnecessary, and I would get that message every day... that was the one frustrating part about the whole thing.*

[Participant ID5]

### Acceptability

#### Overall Satisfaction

**Figure 5.**
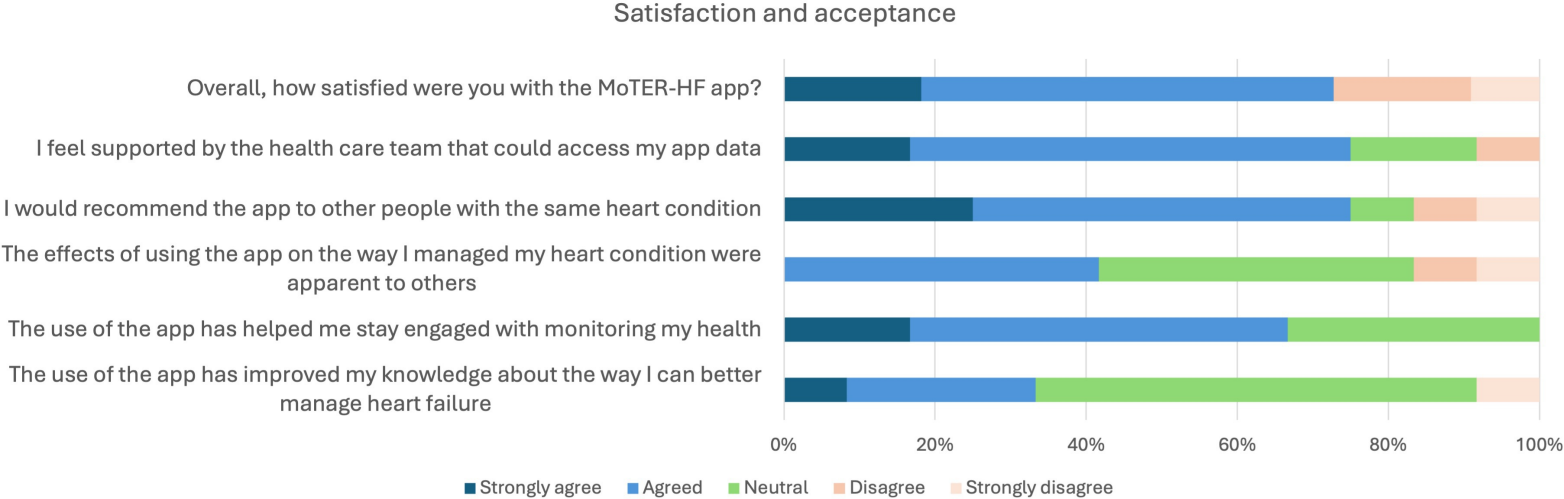
Self-reported satisfaction and perception of empowerment and observability.

Satisfaction questionnaire results indicated that most participants were satisfied with the app (8/11, 72.7%; Figure 5). Participants rated the benefits of improved self-monitoring and support from the health care team highly. These perceptions were consistent with interview findings, in which participants highlighted the app’s role in supporting daily monitoring:

*Overall we liked the idea of being able to actually see a graph of your body weight and your blood pressure that was really good...it covered everything that it needed to cover for a heart failure patient.*

[Participant ID18]

#### Perceived Benefits

##### Improved Self-Monitoring and Self-Management

The majority of participants (8/12, 66.7%) reported in the questionnaire that using the app helped them stay engaged with monitoring their health, although they felt it did not improve their knowledge of heart failure management.

Most interview participants indicated that the app enhanced their motivation to consistently self-monitor health measures, including BP, HR, and weight, compared with their habits before the study. One participant noted that the app had little impact on their monitoring practices, as they had already been tracking these measures regularly before the study.

Some interview participants indicated that the ability to see the data in both graphical and list formats provided them with confidence and encouragement for long-term self-monitoring. A few even expressed interest in keeping the study-provided measurement devices or buying similar BP monitors and digital scales:

*Especially lot of healthcare things you can’t fix overnight, they take months, where if you have the graph...it definitely gives you the confidence that you’re doing the right thing and you’re going the right way.*

[Participant ID5]

Two interview participants indicated that the app helped them manage their heart condition by increased awareness of changes in health measures, such as fluid retention and BP fluctuations. While they did not feel the app would “save their life,” and already knew how to adjust medication for fluid retention or understood symptoms such as dizziness from low BP, they valued the app as an added layer of support for self-managing their heart failure.

##### Awareness of Clinical Nurses Reviewing the Data

Most participants (9/12, 75%) reported in the questionnaire that they were supported by the clinicians who could access their app data.

The majority of interview participants were aware that study nurses could view their app data, which gave them peace of mind. Some mentioned discussing the data with the nurses during their clinic appointments or when the clinical nurse coordinator followed up with them for out-of-range measures. This additional oversight provided a sense of security and made them feel more supported:

*That was really beneficial actually that’s probably one of the strengths of the App we went knowing that for us, knowing that someone else can also see those results and yeah, that was really good.*

[Participant ID18]

##### Sharing Data With General Practitioners

Interestingly, 4 interview participants mentioned sharing their app data with their general practitioners during consultations, finding this helpful for evidence-based discussions. One participant suggested adding a function to generate a summary of the app data, while another highlighted the need for coordinated care if the portal were potentially able to be accessed by multidisciplinary professionals providing different aspects of care:

*When I had issues with lower blood pressure and I went to my local GP, I got the App out and showed him...and he flipped through it...So yes it did help me in that aspect as well.*

[Participant ID5]

##### Receiving Support and Interest From Family Members

The satisfaction questionnaire showed that 41.7% (5/12) of participants felt that the effects of using the app on the way they managed their heart condition were apparent to others, and 75% (9/12) reported that they would recommend the app to others with the same condition. In interviews, 3 participants mentioned that their family members showed support or interest in using the app. One participant, who was less confident with the app, received technical assistance from his wife. Another participant mentioned that his parents, who had chronic health conditions, recognized the app’s potential benefits and expressed interest in using it:

*Because my father has his own kind of conditions, they found it interesting, that it’d be something useful...if they were using a similar application and devices and take that information to their next doctor’s appointment.*

[Participant ID24]

### Health and Behavioral Outcomes 

There were no significant differences in health and behavioral outcome measures compared to baseline, although the “after” results showed improved scores in Self-Care of Heart Failure Index (confidence, maintenance, management, and perception); Depression, Anxiety, and Stress Scale (anxiety and stress); Kansas City Cardiomyopathy Questionnaire (overall, physical limitation, QoL, and symptom frequency); and Self-efficacy for Managing Chronic Disease Scale. Given the small sample size and short study period limitations, these score changes were observations only.

Physical examination records (n=7) showed no significant differences in BP and weight compared to baseline, although the readings were slightly higher at the week 12 follow-up. No Major Adverse Cardiovascular Event associated with participants was reported in the hospital information systems during the study period.

Summary of paired *t* tests for these validated outcome measure questionnaires and selected physical examination measures is presented in Table 4.

**Table 4.** Health and behavioral outcome measurement results^a^.

Outcome measures	n	*P* value	Mean difference (L95% CI, U95% CI)
SCHFI^b^_Confidence	10	.61	− 2.64 (−13.67, 8.40)
SCHFI_Maintenance	10	.12	− 6.00 (−13.81, 1.81)
SCHFI_Management	10	.24	− 5.91 (−16.54, 4.73)
SCHFI_Perception	10	.60	− 3.36 (−17.11, 10.39)
KCCQ^c^_Physical limitation	11	.30	− 9.83 (−29.88, 10.22)
KCCQ_Quality of life	11	.15	− 7.33 (−17.71, 3.05)
KCCQ_Social limitation	11	.60	2.83 (−8.77, 14.44)
KCCQ_Symptom frequency	11	.42	− 3.83 (−13.89, 6.22)
KCCQ_Overall score	11	.30	− 4.50 (−13.52, 4.53)
DHFKS^d^	11	.27	0.50 (−0.46, 1.46)
Self-efficacy^e^	11	.39	− 0.25 (−0.86, 0.36)
DASS21^f^_Anxiety	11	.08	2.83 (−0.39, 6.06)
DASS21_Depression	11	.39	−0.25 (−0.22, 5.55)
DASS21_Stress	11	.23	1.33 (−0.99, 3.65)
Physical exam_Weight	7	.76	− 0.63 (−5.35, 4.10)
Physical exam_Lying diastolic BP^g^	7	.48	− 2.88 (−11.92, 6.17)
Physical exam_Lying systolic BP	7	.27	− 5.00 (−14.77, 4.77)

a Data were filtered to only include those participants who completed both baseline and end-of-study questionnaires. The mean difference is calculated as the sample mean of baseline minus end-of-study values for each participant.

b SCHFI: Self-Care of Heart Failure Index.

c KCCQ: Kansas City Cardiomyopathy Questionnaire.

d DHFKS: Dutch Heart Failure Knowledge Scale.

e Self-efficacy: Self-efficacy for Managing Chronic Disease Scale.

f DASS21: Depression, Anxiety, and Stress Scale-21.

g BP: blood pressure.

## Discussion

### Overview

Despite the growing use of mHealth technologies to support heart failure self-management, challenges remain in understanding patient engagement, the most appropriate features, and how best to evaluate their outcomes and benefits [16,18,20]. This study examined the feasibility of an mHealth intervention developed to support self-management in patients with heart failure. A key strength of this study is its mixed methods design, which provides a comprehensive understanding of patient engagement, user experiences with specific app features, factors impacting engagement, and preliminary potential health outcomes. By analyzing system usage logs, self-reported user experiences from questionnaires, perceptions from in-depth interviews, and health outcome measures, the study offers valuable insights into the app usage patterns, acceptability, facilitators of and barriers to engagement, and the potential impact on patients’ health and self-care behavior. Furthermore, the findings of this study contribute to mHealth design research by demonstrating that feature uptake is driven less by availability or usability and more by alignment with existing care practices, perceived need, and integration within clinical ecosystems.

### Engagement and Acceptance

The study examined engagement patterns associated with individual app features, factors influencing use, and patients’ overall acceptance of the mHealth technology. Research in the literature highlights the importance of unpacking engagement with individual components of mHealth intervention to avoid the “black box” issue of unclear user engagement and effectiveness elements [18]. Consistent with this perspective, our study findings showed that engagement was selective rather than uniform across app functions, offering important insights for the design of mHealth interventions in this patient population.

Participants reported satisfaction and perceived improved self-monitoring practices, with most finding the app easy to integrate into their daily routines and aligned with their preferred self-management practices. Engagement was notably high with core monitoring features, such as BP and weight tracking, due to the alignment with their existing monitoring practices, the Bluetooth-enabled automatic data entry, and the perceived value of visualizing historical data. These findings show that features that seamlessly extend established routines and provide monitoring value are more likely to be sustained over time.

Engagement with other features, such as symptom reporting, exercise tracking, goal checking, and education materials, was lower. Our interview findings suggest that this did not necessarily reflect poor usability but rather limited perceived relevance for participants. Participants described stable health conditions, long-standing self-management experience, or ongoing clinical contact, which reduced the perceived added value of symptom logging, goal review, or educational materials. Exercise tracking was viewed by some participants as redundant with existing rehabilitation programs, which were not digitally integrated with the app, while educational content competed with preferences for direct clinical advice or alternative learning modes.

Our findings suggest that acceptability and adherence to mHealth interventions are maximized when features are aligned with patients’ clinical needs, existing care structure, and personal preferences. Features such as education, exercise tracking, and goal setting may require greater personalization and closer integration with their existing care to enhance perceived value. Studies of mHealth adoption in patients with heart failure have reported person-related, technology-related, and contextual-related engagement barriers [24]. Our findings extend existing evidence and highlight the need for tailoring both content and functionality to the specific circumstances of patients.

From a design perspective, our results align with a growing body of research in mHealth development that emphasizes the value of tailored design and appropriate evidence-based information in a way that is relevant to patients, particularly for older adults and those who require self-management support [23,35]. The initial user testing of the prototype prior to the feasibility study exemplified these principles by incorporating clinician-developed Daily Diary questions and Symptoms monitoring list, as well as clinician-customized daily health measure items. The app provided flexible data entry options of both automatic Bluetooth integration and manual input for smooth use. Participants expressed preferences for varying daily reminder notifications. Some preferred alternative formats for materials in the Education feature of the app, such as audio-based content, which may be a consideration for improving the app design. These findings reinforce the importance of personalization in mHealth design [23,24,35]. Furthermore, technical issues were barriers for some participants, indicating the need for robust systems and user-friendly features to support older patients or those with limited digital experience [23,35].

The implementation of mHealth in real-world settings requires more than just technical design. It also needs effective integration with ongoing clinician follow-ups and existing care programs [22,36]. Our study found that participants felt supported by the study clinicians who could access their data and valued the discussions about their data during face-to-face consultations. However, limited integration with rehabilitation programs and communication pathways reduced engagement with some app features. While some of these limitations may be attributed to the feasibility nature of the study, our findings echo previous research and suggest that incorporating the app data review in outpatient consultations, aligning with patients’ health care programs, and enhancing clinician integration could encourage more consistent use and better support for heart failure self-management [35,37]. Although clinician oversight was identified as a perceived benefit, its sustainability is contingent on balancing clinical value with workload demands when integrating mHealth solutions into clinical workflow [22,36]. Continuous monitoring was not performed in this study due to the limited availability of study nurses and the additional workload it would have had on their existing clinical practices. Technical training and troubleshooting also created additional tasks for study nurses. Furthermore, while workload was not formally measured, the process of reviewing app data and responding to alerts suggests a substantial time commitment that may increase with larger cohorts. Scaling up digital health solutions will require addressing barriers at individual, organizational, policy, and technological levels [36,38]. In larger populations, manual review of patient data and alerts may become unsustainable without automation, prioritization algorithms, and redistribution of responsibilities within care teams. Strengthening system functionality and interoperability will be crucial for transitioning to a scalable digital model of care. Effective implementation will also require strong leadership, supportive regulatory and reimbursement frameworks, and stakeholder cocreation [36,38]. These implementation challenges and strategies will need to be further explored in future studies.

### Health and Behavioral Outcomes

The study also explored changes in health and behavioral outcomes using validated measures and qualitative data. While no statistically significant improvements were observed in self-care, QoL, heart failure knowledge, self-efficacy, and psychosocial measures, exploratory patterns of change consistent with improvement on several subdomains (eg, confidence, maintenance, management, and perception of self-care) were observed. Due to the small sample size and relatively short study period, these changes should be interpreted as observations for guiding future rather than evidence of efficacy. Participants described perceived benefits in managing their condition, as reflected in the satisfaction questionnaire and follow-up interviews, where participants valued improvements in self-monitoring, a better understanding of their health measures, and the sense of reassurance provided through study nurses’ monitoring and discussions, although these perceptions do not imply corresponding measurable changes in health and behavioral outcomes.

In line with recommendations from the broader mHealth literature, longer-term RCTs with larger and more diverse cohorts will be needed to determine whether mHealth platforms, such as MoTER-HF, can produce meaningful improvements in health and behavioral outcomes. Our study results can serve as an important foundation for future studies by providing useful insights into the selection of appropriate outcome measures and the sample size calculations for future RCTs.

### Limitations

The feasibility study had some limitations. The platform used was a research prototype, and troubleshooting during the feasibility study presented challenges. Technical issues, such as problems with Bluetooth device pairing and connectivity, affected participant engagement. Practical barriers, such as scheduling difficulties for participant onboarding and offboarding at their clinic visits and limited time available for the clinical nurse coordinator to follow up with participants, also contributed to missing questionnaire data at the 2 time points. Another limitation was the relatively small number of participants, with participants who were recruited from a single hospital, which might limit the generalizability of the findings.

Future work will focus on gaining a more in-depth understanding of clinicians’ workload and resource requirements, which were not captured in the feasibility study, as only one nurse practitioner and one clinical nurse coordinator who were part of the study team were directly involved in managing the portal and reviewing app data and alerts. Furthermore, following this feasibility study, a larger-scale study within routine clinical workflows will be necessary to evaluate the efficacy and cost-effectiveness of mHealth for heart failure management, as well as to assess the correlations between patient engagement and health outcomes. These efforts will inform the development of a scalable, evidence-based digital health program that can be seamlessly integrated into clinical practices, ensuring its effectiveness and sustainability in real-world settings.

### Conclusions

Overall, the feasibility study demonstrated the potential of MoTER-HF to support self-management in patients with heart failure. Most participants actively engaged with the app for health monitoring and reported positive experiences of usefulness and usability, although no statistically significant improvements in health and behavior measures were observed. The study identified several factors influencing participant engagement and provided insights for the future technical design and implementation. Given that this was a feasibility study, further evidence-based research is needed to investigate its efficacy and to guide the development of an integrated mHealth program and a digital model of care.

## Supplementary material

10.2196/89416 Multimedia Appendix 1 Key features of the MoTER-HF mobile app. From left to right: main menu, Daily Diary, Symptoms, Health Measures (blood pressure data entry), and Exercises.

10.2196/89416 Multimedia Appendix 2 Satisfaction questionnaire.

## Abbreviations

BP: blood pressure
CSIRO: Commonwealth Scientific and Industrial Research Organization
DASS21: Depression, Anxiety and Stress Scale
HR: heart rate
mHealth: mobile health
MoTER: Mobile Technology–Enabled Rehabilitation
QoL: quality of life
RCT: randomized controlled trial
TPCH: The Prince Charles Hospital
