# Supplementary figures and images for: A Mobile Health Platform for Heart Failure Self-Management: Feasibility Study on Patient Engagement, Acceptance, and Potential Health Outcomes
Source: JMIR Form Res. 2026 Jul 10;10:e89416. doi: 10.2196/89416 (PMC13360581; doi:10.2196/89416)

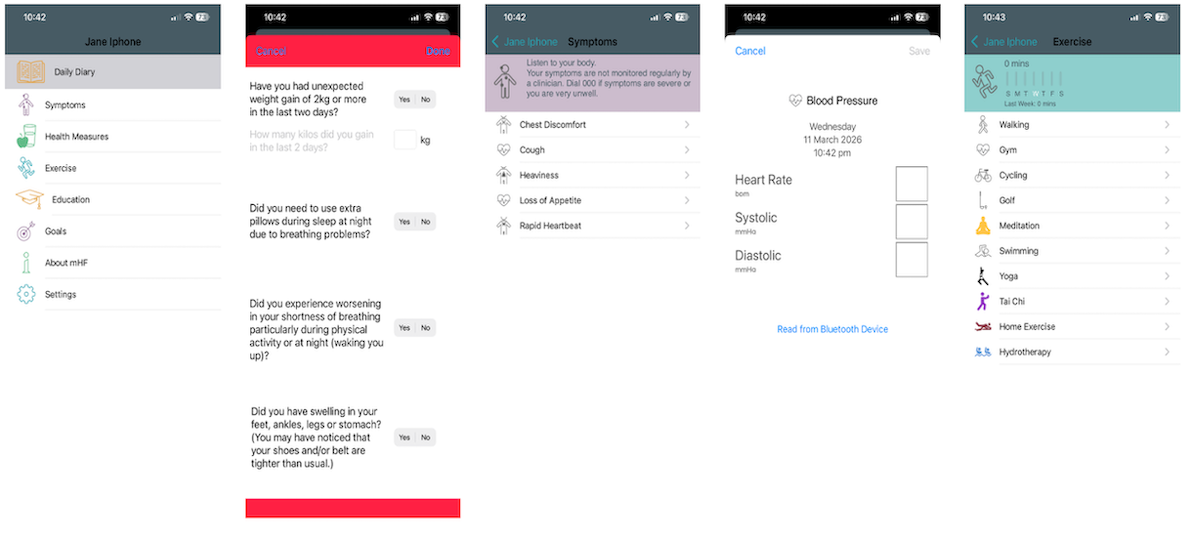

Supplement: Multimedia Appendix 1 [file formative-v10-e89416-s001.png]
