# Supplementary material for: A Mobile Health Platform for Heart Failure Self-Management: Feasibility Study on Patient Engagement, Acceptance, and Potential Health Outcomes
Source: JMIR Form Res. 2026 Jul 10;10:e89416. doi: 10.2196/89416 (PMC13360581; doi:10.2196/89416)
Supplement: Multimedia Appendix 2 [file formative-v10-e89416-s002.pdf]

Satisfaction and Experience Questionnaire

1. Technology Satisfaction

In this section we ask questions about your satisfaction with the MoTER-HF app you have used. Please tick one option for each item.

Usability of the MoTER-HF app	Strongly disagree	Disagree	Neutral	Agree	Strongly agree
1. I think that I would like to use the app frequently.	☐	☐	☐	☐	☐
2. I found the app unnecessarily complex.	☐	☐	☐	☐	☐
3. I thought the app was easy to use.	☐	☐	☐	☐	☐
4. I think that I would need the support of a technical person to be able to use the app.	☐	☐	☐	☐	☐
5. I found the various functions in the app were well integrated.	☐	☐	☐	☐	☐
6. I thought there was too much inconsistency in the app.	☐	☐	☐	☐	☐
7. I would imagine that most people would learn to use the app very quickly.	☐	☐	☐	☐	☐
8. I found the app very cumbersome to use.	☐	☐	☐	☐	☐
9. I felt very confident using the app.	☐	☐	☐	☐	☐
10. I needed to learn a lot of things before I could get going with this app.	☐	☐	☐	☐	☐

2. Self-monitoring and Engagement Experience

In this section, we ask questions about your 12-week self-monitoring and engagement experience – supported by MoTER-HF app with daily health check-ins, goals and education, as well as discussions with clinicians.

Please tick one option for each item.

Empowerment	Strongly disagree	Disagree	Neutral	Agree	Strongly agree
11. The use of the app has improved my knowledge about the way I can better manage heart failure	☐	☐	☐	☐	☐
12. The use of the app has helped me stay engaged with monitoring my health	☐	☐	☐	☐	☐

Engagement	Strongly disagree	Disagree	Neutral	Agree	Strongly agree
13. I was able to engage with the app and maintain my interest to enter the health data as required	☐	☐	☐	☐	☐
14. The ability to review my health data on the app was helpful	☐	☐	☐	☐	☐
15. The health measures set up by clinicians were relevant to my condition	☐	☐	☐	☐	☐
16. The daily diary was useful	☐	☐	☐	☐	☐
17. The ability to record symptoms was helpful	☐	☐	☐	☐	☐
18. The ability to record exercises was helpful	☐	☐	☐	☐	☐
19. The weblinks and heart failure information within the app were useful	☐	☐	☐	☐	☐
20. I was satisfied with the goals developed by clinicians	☐	☐	☐	☐	☐
21. I feel supported by the health care team that could access my app data	☐	☐	☐	☐	☐

Compatibility	Strongly disagree	Disagree	Neutral	Agree	Strongly agree
22. The use of the app was easy to incorporate into my daily routine	☐	☐	☐	☐	☐
23. The use of the app fitted well with my lifestyle	☐	☐	☐	☐	☐
24. The use of the app fitted into the way I liked to manage my health	☐	☐	☐	☐	☐

Observability	Strongly disagree	Disagree	Neutral	Agree	Strongly agree
25. The effects of using the app on the way I managed my heart condition were apparent to others	☐	☐	☐	☐	☐
26. I would recommend the app to other people with the same heart condition	☐	☐	☐	☐	☐

**Overall satisfaction**					
	**Very disappointed**	**Disappointed**	**Neutral**	**Satisfied**	**Very satisfied**
27. Overall, how satisfied were you with the MoTER-HF app?	☐	☐	☐	☐	☐

**Frequency of use**					
28. How often did you use the following functions of the MoTER-HF app to add your data or review information?	**Rarely**	**1-3 times a week**	**4-5 times a week**	**4-6 times a week**	**Daily**
Daily Diary	☐	☐	☐	☐	☐
Symptoms	☐	☐	☐	☐	☐
Health Measures	☐	☐	☐	☐	☐
Exercise	☐	☐	☐	☐	☐
Education	☐	☐	☐	☐	☐
Goals	☐	☐	☐	☐	☐

29. What did you like most about the MoTER-HF app?

----

30. What would you like to change about the MoTER-HF app?

----

31. Please provide any additional comments not covered in the previous questions.

____
